# Recent Advancements in Toxin and Antitoxin Systems Involved in Bacterial Programmed Cell Death

**DOI:** 10.1155/2010/781430

**Published:** 2010-12-27

**Authors:** Ming-xi Hu, Xiao Zhang, Er-li Li, Yong-Jun Feng

**Affiliations:** School of Life Science, Beijing Institute of Technology, Beijing 100086, China

## Abstract

Programmed cell death (PCD) systems have been extensively studied for their significant role in a variety of biological processes in eukaryotic organisms. Recently, more and more researches have revealed the existence of similar systems employed by bacteria in response to various environmental stresses. This paper summarized the recent researching advancements in toxin/antitoxin systems located on plasmids or chromosomes and their regulatory roles in bacterial PCD. The most studied yet disputed *mazEF* system was discussed in depth, and possible roles and status of such a special bacterial death and TA systems were also reviewed from the ecological and evolutionary perspectives.

## 1. Introduction

While the mechanism of autophagy and apoptosis in multicellular eukaryotes has been decoded progressively, many recent researches have suggested that simple prokaryotes like bacteria can also activate particular programmed cell death (PCD), allowing them to survive from the environmental stresses as nutrients deprivation, antibiotics, and so forth. Bacterial PCD has been observed in many bacteria species, including *Escherichia*, *Staphylococcus*, *Pseudomonas, *and *Bacillus *[1]. For eukaryotes, apoptosis plays an important role in embryonic development, immune tolerance and stability, cancer, inflammation, natural regeneration of cells, and other physiological or pathological processes [2]. But for prokaryotes, in essence, PCD is a kind of altruistic act that provides a way to survive the environmental stresses at the expense of some of its cells. This paper will discuss the recent advancement in toxin-antitoxin systems employed by bacteria in their PCD processes and the roles of bacterial PCD from the ecological and evolutionary perspectives.

## 2. PCD Regulated by Toxin and Antitoxin Systems

### 2.1. Toxin-Antitoxin (TA) Systems

For most eubacteria and archaea, the most common mechanism involved in bacterial PCD is the toxin-antitoxin (TA) system. TA systems often include a stable toxin and a labile antitoxin; the former always exists as a stable protein, while the latter is either a protein or an untranslated antisense RNA species. In addition, the antitoxin gene often locates upstream of the toxin gene, usually overlapping or being separated by a small intergenic region, so that TA genes are cotranscribed and, in most cases, cotranslated. The expression of TA operon is autoregulated by antitoxin at the transcription level, while toxin acts as a corepressor [3]. TA systems are often divided into two types [4]: if the toxin is neutralized by an antisense RNA, the system is known as type I [5]; if the toxin is neutralized by avid binding of the partner antitoxin protein, the system is known as type II [6]. Meanwhile, an entirely new TA system has been defined recently, which functions *via* a novel protein-RNA mechanism [7]. During investigation on strategies used by bacteria in resisting bacteriophage infection, the ToxIN abortive infection (Abi) system is demonstrated as a new TA pair, with ToxN inhibiting bacterial growth and the tandem-repeat ToxI RNA antitoxin counteracting the toxicity. Noticeably, the ToxI RNA does not act like type I antitoxin, but is predicted to interact directly with ToxN and inhibit its toxicity.

Recent computational analyses have shown that TA systems are widely presented in eubacterial and archae chromosomes, yet their localizations are quite varied [8]. Based on massive amount of data, researchers have shown that type II TA systems have an extensive horizontal mobility [6]; while type I TA systems are not prone to transfer, but evolved from lineage-specific duplications [9]. Some TA systems are localized within exogenous DNA islands such as phages, transposons, and superintegrons; while others, like *mazEF* from *E. coli* K-12, are settled in the regular positions of the chromosomes [10].

### 2.2. TA Systems Located on Plasmids

The *ccd *(control cell death) system located on the *E. coli* F plasmid is the first TA system discovered [11]. The coexpressed gene products encoded by the *ccd* operon are an unstable antitoxin CcdA that is degraded by the Lon ATP-dependent protease and a toxin CcdB that targets the DNA gyrase (an essential type II topoisomerase) [12, 13]. Under normal growth conditions, the toxic activity of CcdB is inhibited by CcdA by forming a tightly binding iso-dimer [14]. But once the plasmid is lost, the short-lived CcdA will not be able to replenish by the *de novo* synthesis, leaving the long-lived toxin alone in the cytoplasm. As a result, DNA-gyrase complexes are trapped by CcdB, leading to eventually cell death [15]. Related investigations have found that plasmids R1/R100, P1, RK2/RP4, and RSF1010 also possess such type of TA systems, where the toxins interact with DNA helicase [16]. 

Kid/Kis module on *E. coli* plasmid R1 belongs to another kind of TA systems, where toxin plays a role in cleaving RNA and inhibiting protein synthesis [17]. Kid and Kis are the stable toxin and unstable antitoxin encoded by the *parD* operon of plasmid R1 [18]. The* parD* operon is regulated by Kis and Kid coordinately [19]. Kid is a specific endoribonuclease to cleave RNA preferentially at the 5′ side of the adenosine residue in the nucleotide sequence 5′-UA(A/C)-3′ of single-stranded regions. Kis autoregulates *parD* transcription to a limited extent through its N-terminal region [20].

As the genetic units outside the chromosome, plasmids can self-replicate and maintain genetic stability of bacteria. In fact, they are likely to carry essential structural or functional gene sequences that the chromosome genome does not encode. Therefore, it is particularly important for the host to guarantee the stability of these plasmids. Many studies have showed that plasmids tend to have a core area to ensure their replication stability and conjugation transfer. During cell division, plasmid-specified partition proteins are activated. This process is directed by the plasmid itself. Because antitoxin is degraded continuously, the daughter cells that fail to acquire a plasmid copy will result in selective killing or growth impairment [21, 22]. 

As a suicidal system, TA loci only exhibit functions inside cells. This is obviously different from enterobactin or antibiotics, as they are always secreted into extracellular medium to reveal their bioactivity. Most of the well-characterized TA systems resided in plasmids are shown in Table 1.

### 2.3. TA Systems Located on Bacterial Chromosomes

TA systems are also abundant in chromosomes (Table 2) and show more complex phylogenetic patterns. In *E. coli*, there are several pairs of TA modules, including *mazEF *[23, 24], *chpBIK *[25], *relBE *[26, 27], *yefM-yoeB *[28, 29], and *dinJ-yafQ *[30] at least. Schmidt et al. [31] found that *prlF *and *yhaV *encode a new toxin-antitoxin system in *E. coli*. As homologs of MazE and RelE, respectively, PrlF and YhaV provide an evolutionary connection between the two best-characterized* E. coli* TA systems (*mazEF* and *relEB*). Besides the TA systems described in Table 2, three new chromosomal TA systems Rv1246c-Rv1247c, Rv2865-Rv2866, and Rv3357-Rv3358, named as RelBE, RelFG, and RelJK, respectively, were identified in *Mycobacterium tuberculosis* recently [32].

While most TA modules conform to the characteristic cassette, in which the antitoxin gene precedes the toxin gene as mentioned above, there are also examples that the gene order is reversed, or the product of a third gene is implicated [21]. A recent study by Nariya and Inouye demonstrated that *Myxococcus xanthus* has a solitary *mazF *gene that lacks a cotranscribed antitoxin gene *mazE *[33]. The deletion of *mazF* causes elimination of the obligatory cell death during development, resulting in dramatic reduction in spore formation. Surprisingly, a key developmental regulator, MrpC, is discovered to function as a MazF antitoxin and a *mazF* transcription activator. The transcription of *mrpC* and *mazF* is negatively regulated* via* MrpC phosphorylation by a Ser/Thr kinase cascade.

In conclusion, toxin-antitoxin systems are ubiquitous in both bacterial chromosomes and plasmids. So far, most researches have focused on Gram-negative bacteria, especially in *E. coli*; in contrast, information on Gram-positive bacteria is quite limited. Discoveries include the *relBE* and *mazEF* systems in *Streptococcus mutans* and enterococci [34, 35], the *talAB* system in *Leifsonia xyli *[36], the Rv1102c-Rv1103c system in *Mycobacterium tuberculosis* [37], and so forth. Further examples are shown in Tables 1 and 2. These TA systems are suggested to have similar functions to their homologues in Gram-negative bacteria.

## 3. *mazEF* System and Bacterial PCD

As the best studied bacterial PCD module, *mazEF* system has the following features: (i) MazF is toxic and MazE is antitoxic [38]. MazF is a sequence-specific endoribonuclease, which cleaves mRNA between A and C at the ACA sequence [39, 40]; (ii) MazF is long-lived, whereas MazE is a labile protein degraded *in vivo* by the ATP-dependent ClpPA serine protease [41]; (iii) MazE and MazF have the ability to associate into MazF_2_-MazE_2_-MazF_2_ hetero-hexamers in physical conditions [42]; (iv) MazE and MazF are coexpressed, located downstream the *relA* gene in the *rel* operon [41]; (v) *mazEF* is negatively autoregulated at the transcription level by the combined action of both MazE and MazF proteins [43].

Several investigations have revealed that some stress conditions, including starvation, antibiotics, high temperature, DNA damage, and oxidative stress, can trigger bacterial cell death through *mazEF* system [24, 44, 45]. Guanosine 3′, 5′-bispyrophosphate (ppGpp) has been revealed to regulate the starvation-triggered PCD [41]. As the key signal molecule signs of amino acids deficiency, the synthesis of ppGpp in *E. coli* is governed by two pathways. One is activated by amino acid deprivation. The enzyme responsible for this pathway is encoded by *relA*, which is induced by uncharged tRNA or inhibited by amino acylation. The other pathway is activated by the limitation of carbon source, which is *relA*-independent [81]. It is indicated that under nutritional starvation conditions, increased ppGpp will then inhibit the coexpression of *mazE* and *mazF*. As MazE is decreased rapidly, MazF can exert its toxic effect and cause cell death. Artificial overproduction of ppGpp could by itself cause *mazEF*-dependent cell death, providing an experimental evidence for the model above [41]. 

Bacterial PCD mediated by *mazEF* is also a population phenomenon. Antibiotics inhibiting transcription and/or translation (such as rifampicin and chloramphenicol) can prevent the synthesis of the short-lived antitoxin MazE and then induce PCD in *E. coli* [44]. This interesting finding leads to the discovery of a novel linear pentapeptide quorum sensing signal (an extracellular death factor, EDF): Asn-Asn-Trp-Asn-Asn (NNWNN) [82]. This signal peptide is essential for *mazEF*-mediated cell death [83], and thus necessary for reactive oxygen species (ROS) production [84]. Previous studies have confirmed that antibiotics inhibiting transcription and/or translation can cause *mazEF*-mediated PCD by forming ROS. Things are different for other antibiotics that cause DNA damage (like nalidixic acid); they also induce ROS production, however, arouse *mazEF*-mediated PCD through an ROS-independent way [85]. Further investigation by Amitai et al. discovered several “survival proteins” that are only triggered in the ROS-dependent pathway [86]. This provides a perspective in understanding how bacteria deal with different stress conditions and how to choose between living and death. 

**Figure 1:**
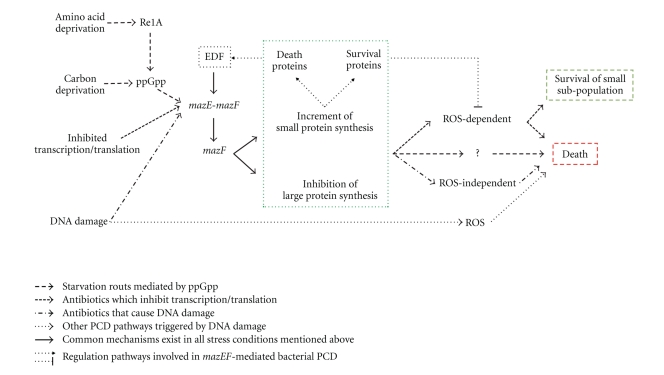
Mechanisms involved in *mazEF*-mediated bacterial PCD. *mazEF*-mediated bacterial PCD can be triggered by several stress conditions including starvation, antibiotics, DNA damage, and so forth.

Mechanisms involved in *mazEF*-mediated PCD are briefly summarized in Figure 1. It is clear to see that EDF and ROS are the crux of the matter. Kolodkin-Gal et al. [82] searched the entire *E. coli* genome for DNA sequences encoding the amino acid sequence of NNWNN and found that EDF most likely comes from the modification of glucose-6-phosphate dehydrogenase (G6PD). G6PD is a key metabolic enzyme to catalyze the first and rate-limiting step of the pentose phosphate shunting and produce nucleotide precursors [87]. Even more interestingly, as oxidative stress is the major death inducer in both prokaryotes and eukaryotes, the role of G6PD in diminishing oxidative stress and protecting mammalian cells from apoptosis has been reported recently, and ROS modulation is essential for cytosolic cytochrome C to fully activate caspases and apoptosis [88]. Therefore, it may be reasonable to infer that G6PD might also play an important role in bacterial PCD regulation, and that EDF generated from G6PD may link metabolism and PCD through ROS.

## 4. PCD Related to Bacterial Biofilm

As structured multicellular communities, biofilms can be found in diverse environments. Biofilm strategy is adopted to protect bacteria from many environmental stresses (e.g., antibiotics, UV lights, or heavy metal toxicities), realize better use of nutrient, acquire metabolic cooperativity, and even obtain new genetic traits [89]. Researchers have demonstrated that cells at different zones of biofilm show different metabolic activities, with those embedded in the center having the lowest activity [90, 91]. Many recent studies have suggested that bacterial PCD played a significant role in biofilm formation and development [92, 93]. In such a structure, PCD may have multiple functions like (i) creating water channels within biofilms to transport nutrients or waste into and from cells deeply inside; (ii) releasing extracellular DNA that can be used for structuring biofilms [94]; and (iii) allowing cells to dispersal from the biofilm matrix when bacteria need to escape the architecture [95]. 

Although cell death and lysis are definitely linked to biofilm formation [92, 93, 96], reports on the effect of TA systems on biofilm formation are limited. Tsilibaris et al. constructed a mutant *E. coli* strain MG1655 that lacks the five best-studied proteic TA systems (Δ5, MazF/MazE, RelE/RelB, ChpB, YoeB/YefM, and YafQ/DinJ) and found no significant difference between wild type and Δ5 under various PCD-inducing conditions [97]. This result raised a big question on the real function of TA system located on chromosomes. Using these two strains and *E*.* coli *BW25113, Kim et al. investigated whether these five TA loci affect biofilm formation. They found that compared to MG1655 WT, Δ5 decreased biofilm formation at 8 h and increased biofilm formation at 24 h. This result is caused by YjgK overexpression, a protein affecting the production of fimbriae involved in both biofilm attachment and dispersal. Furthermore, a toxin Hha and an antitoxin YefM may be involved in biofilm formation. In fact, deletions of each of the five toxins and overexpressions in another genetic background strain BW25113 have totally opposite results in biofilm formation [95]. Then, it is interesting to notice that as Kolodkin-Gal and Engelberg-Kulka described recently, unlike many well-studied *E*.* coli* strains, strain MG1655 used in the experiments mentioned above is partially defective both in production and response to EDF; thus *mazEF*-mediated cell death does not occur [83]. Following this result, they further studied how each of these five TA systems affected bacterial cell death differently during biofilm formation in *E. coli* strain MC4100 [98]. Their findings are as follows: (i) *mazEF* is the regulating module mediating cell death both in liquid media and in biofilm formation; (ii) *relBE* seems to be a principal mediator of cell death only in liquid media, but not in biofilm formation; (iii) *chpBIK* seems to be a back-up death system for *mazEF* in a ppGpp-independent way; (iv) *yefM*-*yoeB* mediates cell death only in liquid media at some conditions, while not at all in biofilm formation; (v) *dinJ*-*yafQ* seems to be a principal mediator of cell death only in biofilm formation. Compared with previous studies on MG1655 that can be seen as phenotypically MazEF^−^, it is tempting to infer that many TA systems might connect each other as a network where *mazEF* plays as a kind of effector to activate death path under some conditions, while some other TA loci may form alternative regulatory pathways to deal with other stresses. 

This speculation feels more possible when the studies go further to the TA systems connected with persister cell formation, which is a small fraction of bacteria that resist to antibiotics without genetic change forming during the stationary-phase or in biofilm. Kim et al. found that MqsR and MqsA of *E. coli* are toxin-antitoxin pair that influences cell physiology (e.g., biofilm formation and motility) *via* RNase activity as well as through regulation of toxin CspD [69]. They identified eight genes (*cspD*, *clpX*, *clpP*, *lon*, *yfjZ*, *relB*, *relE, *and *hokA*) related to MqsR toxicity and discovered that toxins CspD, Hha, and HokA influence persister cell formation *via* MqsR and small RNA regulator Hfq [99]. Besides, it is interesting to notice that (i) ClpXP is an important protease system for stress-induced environments and degrades RpoS and Dps; (ii) many antitoxins, including MazE and RelB, are degraded by the ATP-dependent ClpP and Lon proteases (Tables 1 and 2); (iii) Besides normally taking place during exponential growth, *mazEF*-mediated cell death also occurs in a Δ*rpoS* mutant at stationary phase, which means RpoS is responsible for the resistance to *mazEF*-mediated PCD during stationary growth [100]; (iv) *hha *is induced dramatically in biofilm. According to the studies on MG1655 and Δ5, the ability of Hha to reduce biofilm formation is dependent on the activity of some of these five TA systems [95]. Furthermore, MqsR is involved in the regulation of motility signaled by quorum sensing factor autoinducer-2 (AI-2). Based on results mentioned above, it is meaningful to investigate TA systems from an interactive view.

## 5. The Possible Roles of Chromosomal TA Systems

Inspecting entire history of biological evolution, although it is filled with competitions among individuals and species, it is undeniable that collaborations still exist. In particular, some altruistic behaviors like PCD were found in unicellular organism (such as bacteria) in recent years. As we mainly discussed above, a group of researchers suggested that at least some of the TA systems act as apoptotic tools. Bacteria can obtain a lot of benefit from this function, as Engelberg-Kulka et al. have pointed out there are three possible functions for PCD systems [101]. Firstly, under nutritional starvation conditions, the death of a part of bacterial individuals can provide food for the survivals to maintain the species. Secondly, PCD acts as a defense approach to prevent the spreading of phage infection, for example, PCD mediated by *mazEF* can prevent the spreading of phage P1 [23]. Thirdly, PCD can also act as a guardian of the bacterial chromosome. When other systems fail, PCD systems might maintain genomic stability by elimination of deficient cells and/or mutations from the culture. The so-called “PCD hypothesis” is represented primarily by *mazEF* module.

However, question marks are still raised on the functions of chromosomal toxin-antitoxin systems. As an alternative hypothesis, another group of scientists [49, 102] suggested that rather than killing the cell, TA systems let the bacteria enter a latent state from which they can recover under favorable conditions and function as metabolic regulators. Take *relBE* for an example: RelE is a kind of mRNA interferases that must cleave mRNA at the ribosomal A site, which is translation-dependent. When bacteria facing nutritional deprivation or other stressful conditions, RelE becomes active and reduces the global rate of translation by mRNA cleavage, causing the cell growth arrest [67], and when the “difficult time” has passed, newly synthesized RelB neutralizes the toxin, letting the cell return to the normal life [103]. First suggested by Pedersen et al. [102], this hypothesis was then challenged by Amitai et al. [38] showing the existence of a no return point in MazF lethality that occurs sooner in minimal growth medium than in rich medium. The point of no return in the action of MazF was further confirmed by Kolodkin-Gal and Engelberg-Kulka [104]. However, many other TA systems, including *relBE*, are still proved to be bacteriostasis. More information has been provided in several excellent reviews [105–107].

Besides, some authors believe that these two different assumptions are not completely incompatible. They suggested that TA systems may be activated by stress and bring cells into stasis. However, escaping from that state may be different in various cells: some of the population may die in the course of transition, while some “lucky beggars” may obtain nutrition at the expense of their less lucky neighbors [107]. 

The existence of problems and controversies reflects the complexity of the TA systems and their versatility of the functions. Recently, at least nine possible functions in chromosome TA systems have been summarized, including junk, stabilization of genomic parasites, selfish alleles, gene regulation, growth control, persisters [108], programmed cell arrest and the preservation of the commons, programmed cell death, antiphage, and so forth [109, 110]. Each chromosome TA system has been evaluated to take part in at least one of the functions. These functions are undoubtedly significant to bacterial collaborative behaviors. The death of a subpopulation may enable to provide construction matrix components (such as the extracellular genomic DNA) for others to form biofilm or persister cell, or to give up some nutrients for others to generate a metabolic activity. This may again draw attention on the speculation we mentioned above: each TA system may deal with different problems through different aspects, and they collaborate with each other, form an exquisite network, to help the species survive. 

## 6. Perspectives

The process of bacterial PCD is an interesting problem, which on the first sight seemed to be a paradoxical phenomenon as the final purpose is survival not death. At present, it is far from clear on this complex physiological process. The remaining questions are how bacteria make the most beneficial choice between living and death, what pivotal death factors in different bacterial groups are, what happens under the direction of the death signals in this process, especially when biofilm formation is involved. 

Another question is that although both bacterial PCD and apoptosis are deeply explored, whether they have evolutionary connection is not clear enough. In fact, by comparing the apoptosis with the bacterial PCD, a significant similarity is found between them [111]. For example, the mechanism of toxin/antitoxin mentioned above is similar to the IAPs/caspase (IAPs is a family of proteins that can inhibit caspase function by binding of their IAP repeat domain to the caspase active site, promoting the degradation of active caspases, or sequestering the caspase away from its substrates) in* Drosophila melanogaster *[112]. This similarity might suggest a common ancestor of those systems, providing us some novel perspectives for the further study. 

Furthermore, the potential value of bacterial PCD in disease control is great. Bacterial PCD provides us an absolute new idea in designing more efficient “antibiotics” [84, 85, 101], which can activate bacterial suicide modules and cause bacterial death in a more special manner. This kind of achievement surely will produce far-reaching effects on human health and present an attractive researching field for the modern biochemists.

## Figures and Tables

**Table 1:** Properties of well-characterized TA systems in plasmids.

TA system	Organism	Toxin	Antitoxin		Target	Protease cleaving the antitoxin	Reference
			Protein	RNA			
*ccdAB*	*E*.* coli *	CcdB	CcdA		DNA gyrase	Lon	[46, 47]
*dinJ-yafQ*	*E*.* coli *	YafQ	DinJ		Unknown	Unknown	[48]
*hok/sok*	*E*.* coli *	Sok		Hok	Cell membrane	Unknown	[49, 50]
*kid/kis*	*E*.* coli *	Kid	Kis		mRNA cleavage	Lon	[51]
*parDE*	*E*.* coli *	ParE	ParD		DNA gyrase	Unknown	[52]
*pemKI*	*E*.* coli *	PemK	PemI		DnaB	Lon	[53]
*relBE*	*E*.* coli *	RelE	RelB		mRNA cleavage	Unknown	[16]
*yefM-yoeB*	*E*.* coli *	YoeB	YefM		mRNA cleavage	Unknown	[54]
*relBE2*	*S. pneumoniae*	RelE	RelB		Unknown	Unknown	[28]
*mazEF*	enterococci	MazF	MazE		mRNA cleavage	ClpPA	[34]
*higBA*	*V. cholerae*	HigB	HigA		mRNA cleavage	Unknown	[55, 56]
	*P*.* vulgaris *						
*phd-doc*	*V. cholerae*	Phd	Doc		30S ribosomal subunit	ClpXP	[57]
*toxIN*	*E*.* carotovora *	ToxN		ToxI	Unknown	Unknown	[7]
*axe-txe*	*E. faecium*	Txe	Axe		Unknown	Unknown	[58]
**ω*-*ε*-*ζ**	*E. faecium*	*ζ*	*ε*		Transcription	Unknown	[59, 60]
	*S. pyogenes*						
*fst*	*E. faecalis*	Fst(RNA I)		RNA II	Translation	Unknown	[61, 62]
	*B. subtilis*						
*mvpTA*	*Sh*.* flexneri *	MvpT	MvpA		Unknown	Unknown	[63]

**Table 2:** Properties of TA systems in chromosomes.

TA system	Organism	Toxin	Antitoxin		Target	Protease cleaving the antitoxin	Reference
			Protein	Antisense RNA			
*ccdAB*	*E*.* coli *	CcdB	CcdA		DNA gyrase	Lon	[64]
*chpBIK*	*E*.* coli *	ChpBK	ChpBI		mRNA cleavage	Unknown	[25]
*dinJ-yafQ*	*E*.* coli *	YafQ	DinJ		mRNA cleavage	Unknown	[30, 65]
*hipBA*	*E*.* coli *	HipA	HipB		Unknown	Unknown	[66]
*mazEF*	*E*.* coli *	MazF	MazE		mRNA cleavage	ClpPA	[23]
*prlF-yhaV*	*E*.* coli *	YhaV	PrlF		Unknown	Unknown	[31]
*relBE*	*E*.* coli *	RelE	RelB		mRNA cleavage	Lon	[67]
*istR-tisB*	*E*.* coli *	TisB		IstR-1	Cell membrane	Unknown	[68]
*mqsA-mqsR*	*E*.* coli *	MqsR	MqsA		RNA cleavage	Lon, ClpXP	[69]
*hicBA*	*E*.* coli *	HicA	HicB		mRNA cleavage	Lon	[70]
*yafN-yafO*	*E*.* coli *	YafO	YafN		mRNA cleavage	Unknown	[71]
*yefM*-*yoeB *	*E*.* coli*, *S. pneumoniae *	YoeB	YefM		mRNA cleavage	Unknown	[54]
*relBE2*	*S. pneumoniae*	RelE	RelB		Unknown	Unknown	[28]
*pezTA(*ε*-*ζ* like)*	*S. pneumoniae*	PezT	PezA		Transcription	Unknown	[72]
*ydcDE*	*B. subtilis*	YdcE	YdcD		Translation?	Unknown	[73]
*pemKI*	*B. anthracis*	PemK	PemI		DnaB	Lon	[74]
*phd-doc*	*N*.* europaea *	Phd	Doc		Translation	ClpXP	[75]
*parDE*	*N*.* europaea *	ParE	ParD		DNA gyrase	Unknown	[75]
*rv1991ac*	*M. tuberculosis*	Rv1991a	Rv1991c		Unknown	Unknown	[76]
*vapBC*	*M. smegmatis*	VapC	VapB		mRNA cleavage	Unknown	[77, 78]
*higBA*	*V. cholerae*	HigB	HigA		Unknown	Unknown	[79]
*mosA-mosT*	*V. cholerae*	MosT	MosA		Unknown	Unknown	[80]
*rv1102c-1103c*	*M. tuberculosis*	Rv1102c	Rv1103c		mRNA cleavage	Unknown	[37]
*talAB*	*L. xyli*	TalB	TalA		Unknown	Unknown	[36]
